# Visual outcomes of observation, macular laser and anti-VEGF in diabetic macular edema in type 1 diabetes: a real-world study

**DOI:** 10.1186/s12886-022-02482-z

**Published:** 2022-06-09

**Authors:** Joonas Wirkkala, Anna-Maria Kubin, Pasi Ohtonen, Joona Yliselä, Taru Siik, Nina Hautala

**Affiliations:** 1 Department of Ophthalmology, University of Oulu, Oulu University Hospital, P.O. Box 21, 90029 Oulu, OYS Finland; 2 PEDEGO Research Unit, University of Oulu, Oulu, Finland; 3 Medical Research Center, University of Oulu, Oulu, Finland; 4 Research Service Unit, Oulu University Hospital, Oulu, Finland; 5 Research Unit of Surgery, Anesthesiology and Intensive care, University of Oulu, Oulu, Finland

**Keywords:** Type 1 diabetes, Diabetic macular edema, Anti-VEGF agents, Diabetic retinopathy

## Abstract

**Background:**

The treatment for diabetic macular edema (DME) has revolutionized during the last 15 years after the introduction of intravitreal anti-VEGF agents. The aim of the current study is to evaluate the real-world visual outcomes of diabetic macular edema (DME) treatment in patients with type 1 diabetes (T1D) in long-term follow-up.

**Methods:**

A real-world, descriptive, population-based cohort and follow-up of all patients with T1D and DME in 2006-2020 in 34 communities of the Northern Ostrobothnia Hospital District. The main outcome measures included age, gender, duration of T1D at the onset of DME, stage of retinopathy, treatment of DME (observation, laser, intravitreal treatments, combination), and visual outcomes.

**Results:**

A total of 304 eyes of 206 T1D patients with DME were included. 75% (*n*=155) had non-proliferative diabetic retinopathy during the onset of DME. 15% of the cases were observed, 33% had macular laser, 41% intravitreal anti-VEGF and 12% combination of laser and intravitreal injections. Patients in anti-VEGF and in combination groups gained 4.9 and 5.5 ETDRS letters after the initial DME episode (*p*<0.001 and *p*<0.001), and the long-term visual improvements were 4.1 and 5.1 ETDRS letters (*p*<0.001 and *p*<0.001), respectively. In observation and laser groups the initial gain of 0.1 (*p*>0.90) and loss of 0.4 ETDRS letter (*p*=0.61), respectively, was noted. After the follow-up, a 3.7 ETDRS letter decrease was documented in the observation group (*p*>0.90) and a 1.1 (*p*=0.14) ETDRS letter decline in the laser group of patients. At the beginning of treatment, eyes subjected to anti-VEGF alone or in combination with laser had lower visual acuity compared to eyes subjected to observation or macular laser. The average of a 6.1±4.8 anti-VEGF injections were needed to dry DME. Visual impairment due to DME decreased from 2.4% to 1.0% during the 15-year period.

**Conclusions:**

Anti-VEGF alone or in combination with macular laser seems to be beneficial in terms of visual outcomes and treatment stability in T1D patients with central DME.

Moreover, satisfying long-term visual outcomes were achieved with anti-VEGF treatment in a real-world setting.

## Background

A recent study has estimated a 0.5% national prevalence of type 1 diabetes (T1D) among adults in the US, which accounted for 5.6% of all diagnosed diabetes [1]. The global prevalence of any diabetic retinopathy (DR) in T1D has been estimated to be 77% and that of proliferative diabetic retinopathy (PDR) to be 32% in a recent meta-analysis [2]. A 4-12% prevalence of diabetic macular edema (DME) in patients with T1D has been reported [3].

The number of visual impairment due to DR has decreased during the past decades despite the increasing number of diabetes cases [4, 5]. More effective screening for DR, the development of the non-invasive optical coherence tomography (OCT) in the diagnosis of DME, introduction of intravitreal administration of anti-vascular endothelial growth factor (anti-VEGF) agents, as well as improvements in the management of diabetes, have been the key factors in the reduction of visual impairment [6, 7]. Lately, DME rather than PDR has been the increasingly common cause of visual impairment [5].

Anti-VEGF agents are currently widely used in treatment of DME and considered the first-line treatment in clinically significant DME [8]. Despite the great number of previous studies evaluating anti-VEGF treatment in DME during the last decade, there are, to our knowledge, no studies reporting long-term real-world DME treatment outcomes solely in patients with T1D. It has been established that the clinical features and risk factors for DME might vary in patients with T1D from patients with T2D [9].

The aim of the current descriptive population-based cohort and follow-up of all patients with T1D and DME is to evaluate DME outcomes in a real-world setting and to explore the effect of the choice of treatment (observation, laser, intravitreal anti-VEGF or combination of laser and anti-VEGF) on long-term visual outcomes. Also, the number of intravitreal injections needed to treat DME, and the rate of DME-related visual impairment are studied.

## Methods

This work was carried out at the Oulu University Hospital. The study followed the tenets of the Declaration of Helsinki and it was conducted with the approval of the Oulu University Hospital Research Committee (175/2016). Involvement of patients or the public in the design, or conduct, or reporting, or dissemination plans of the current research was not appropriate. A written informed consent was obtained from the participants.

This retrospective population-based cohort study was performed on all adult patients with T1D, who presented at the Oulu University Hospital with DME between June 1, 2006 and Dec 31, 2020. All cases of DME observed or treated by laser, intravitreal anti-VEGF or corticosteroid injections or vitrectomy in the Northern Ostrobothnia Hospital District were included in the study. The hospital’s electronic patient database was used to search for the T1D patients with DME by using the ICD-10 (International Classification of Diseases) diagnosis codes for diabetic maculopathy (H36.1) and T1D (E10.3). Optical coherence tomography (OCT) was used to demonstrate the intra- or subretinal fluid in the macula, and a central retinal thickness of ≥ 300 μm was considered as clinically significant macular edema. Demographic data was collected and included parameters for age, gender, age at the diabetes onset, duration of the diabetes at the onset of both DR and DME, stage of DR during the onset of DME, treatment of DR and DME (observation, laser, intravitreal treatments, vitrectomy) and the time of the follow-up. The best corrected visual acuity (BCVA) was evaluated by the Snellen chart during the DME treatment and follow-up. The BCVA results were converted to ETDRS (Early Treatment of Diabetic Retinopathy Study) letters to calculate visual outcomes. The severity of DR was based on the 5-scale classification system by Finnish Current Care Guidelines of Diabetic Retinopathy [10]. For the use of laser or intravitreal treatment, the current protocols, and guidelines of DME treatment were adhered to [8, 10]. As a rule, the patients with an extrafoveal macular edema located ≥ 500 μm from the central fovea were either observed or subjected to macular laser depending on the location and amount of intraretinal or subretinal fluid. Patients with a central macular edema within 500 μm from the fovea were primarily subjected to intravitreal anti-VEGF treatment. The patients were subjected to combination treatment with anti-VEGF and macular laser in cases of subsequent central and extrafoveal edema. The recurrence of DME was considered if there was no intraretinal or subretinal fluid in the macular OCT between the separate episodes of DME. In Finland, bevacizumab is commonly used as the first-line intravitreal drug for DME.

SPSS (IBM Statistical Analysis) version 27.0 was used for the statistical analysis of the data. Summary statistics are presented as mean with standard deviation (SD) unless otherwise stated. Linear mixed model (LLM) was used to compare the change in vision from baseline to the end of follow-up, where the patient and the eye were set as random effect factors to handle intra- and interindividual correlation, and sex, age and time from the diagnosis of T1D as adjusting factors. Mean change with a 95% confidence interval is presented as the result of LLM. Two-tailed *p*-values <0.05 are considered as statistically significant.

## Results

A total of 206 patients (304 eyes) with T1D and DME were included in the population-based study cohort, out of which 121 (59%) were males. The average age at the time of diagnosis of T1D was 23.4土16.5 years (range 1-79 years). The duration of diabetes at the diagnosis of any DR was 16.4土9.7 years (range 0-53 years) and that of DME 24.0土11.9 years (range 0-53 years). The average age of the patients at the onset of DME was 47.4土14.4 years (range 18-85 years). 155 patients (75%) had non-proliferative DR (NPDR) during the time of diagnosis of DME and only 51 patients (25%) had developed proliferative diabetic retinopathy (PDR). During the study period, there were 304 initial episodes of DME in 206 patients with T1D. Including the recurrences, a total of 456 episodes of DME in 304 eyes were noted. The average follow-up time of a single patient was 65土45 months (range 4-236 months). 18 patients deceased (9%) during the 15-year study period. The demographics are presented in Table 1.

**Table 1:** Characteristics of the participants

Patients, n (%)	206
DME in both eyes	98 (48)
Males	121 (59)
Follow-up time, months, mean, SD [min-max]	65.2 (44.9) [6-235]
DR severity at the onset of DME, n (%)	
Mild non-proliferative	31 (15)
Moderate non-proliferative	98 (47)
Severe non-proliferative	26 (13)
PDR	51 (25)
Age at DM, years, mean, (SD) [min-max]	23.4 (16.5) [1-79]
Age at DME, years, mean, (SD) [min-max]	47.4 (14.4) [18-85]
Time from T1D to DR, years, mean, (SD) [min-max]	16.4 (9.7) [0-53]
Time from T1D to DME, years, mean, (SD) [min-max]	24.1 (11.8) [0-59]

*DME* Diabetic macular edema, *DR* Diabetic retinopathy, *PDR* Proliferative diabetic retinopathy, *T1D* Type 1 diabetes

In a total of 45 DME episodes (15%), the patients were observed without treatment for DME. The patients had macular laser treatment in 100 (33%), intravitreal anti-VEGF in 124 (41%) and combination of laser and intravitreal injections in 35 (12%) cases of DME. During the follow-up period between the years 2006-2020, the use of intravitreal agents has significantly increased, after established treatment practices have become more common after the first years of availability of these drugs.

An average baseline BCVA at the time of diagnosis of DME was 76.9土12.5 ETDRS letters (range 15.1-95.2 ETDRS letters). At the onset of DME, five T1D patients (2.4%) were visually impaired according to classification of visual impairment by the World Health Organization (WHO). However, treatment of DME improved the BCVA in several cases, and at the end of follow-up, only two of these patients (1.0%) met the criteria of visual impairment.

Improvements of BCVA of 4.9 (*p*<0.001) and 5.5 (*p*<0.001) ETDRS letters were documented in the anti-VEGF and combination (anti-VEGF and laser) groups after the primary episode of DME, respectively. In addition, patients treated with either anti-VEGF agents alone or in combination with laser, gained statistically significant long-term improvements of 4.1 (*p*<0.001) and 5.1 (*p*<0.001) ETDRS letters, respectively. After the initial DME, there was no significant change in BCVA in the observation group (gain of 0.1 ETDRS letters, *p*>0.90) or in the laser group (decrease of 0.4 ETDRS letters, *p*=0.63). A 3.7 letter decrease was documented in the observation group (*p*>0.90) and a 1.1 (*p*=0.14) letter decrease in the laser group after the follow-up. The visual outcomes of the alternative treatments of DME are presented in Table 2.

**Table 2:** Ophthalmological outcomes

	All *N* = 304	Observation *N* = 45	Macular laser *N* = 100	Anti-VEGF *N* = 124	Macular laser and anti-VEGF *N* = 35
Age at DME onset**, mean (SD)	47.4 (14.4)	47.5 (16.1)	43.0 (12.1)	49.9. (14.5)	44.7 (13.2)
Given anti-VEGF-injections, mean (SD^a^)				6.0 (4.2)	6.5 (6.5)
Visual impaired eyes, n (%)					
at DME onset	22 (7.2)	4 (8.9)	3 (3.0)	11 (8.9)	4 (11.4)
after the first DME	16 (5.3)	3 (6.7)	4 (4.0)	7 (5.6)	2 (5.7)
at the end of follow-up	7 (2.3)	2 (4.4)	3 (3.0)	2 (1.6)	0 (0)
Recurrence of DME, n (%)	193 (63.5)	41 (91.1)	59 (59)	80 (64.5)	13 (37.1)
BCVA , mean (SD^a^)					
at DME onset	76.4 (12.5)	81.6 (8.8)	80.8 (13.1)	72.8 (11.1)	72.4 (13.2)
at the end of first episode	79.7 (11.5)	80.8 (12.6)	80.7 (13.7)	78.9 (9.9)	78.5 (8.9)
at the end of follow-up	78.9 (12.2)	78.3 (17.7)	80.2 (13.4)	78.3 (10.1)	78.7 (9.0)
ETDRS letters gain after first episode, mean (95% CI^b^)	2.9 (2.1 to 3.8)	0.1 (-3.6 to 3.8)	-0.4 (-1.9 to 1.1)	4.9 (3.9 to 6.0)	5.5 (2.9 to 8.1)
*p*-value	<0.001*	>0.90	0.61	<0.001*	<0.001*
ETDRS letters gain at the end of follow-up, mean (95% CI^b^)	1.8 (1.0 to 2.7)	-3.7 (-7.4 to 0.04)	-1.1 (-2.7 to 0.4)	4.1 (3.1 to 5.2)	5.1 (2.5 to 7.8)
*p*-value	<0.001*	>0.90	0.14	<0.001*	<0.001*

*VEGF* Vascular endothelial growth factor, *DME* Diabetic macular edema, *BCVA* Best corrected visual acuity with ETDRS letters

^*^Statistical significance (*p*-value < 0.05)

^**^No statistical significance between groups (*p* = 0.38, ANOVA)

^a^Standard deviation

^b^95% confidence interval

A great majority, over 99%, of the DME patients treated with intravitreal agents, received bevacizumab as a first-line drug. In only a single case (0.6%), aflibercept was used as a primary choice for anti-VEGF treatment. In 15% (*n*=24) of the cases, the switch from bevacizumab to aflibercept was documented during the follow-up. An average of 6.1土4.8 injections of either anti-VEGF drug (6.0 in anti-VEGF group and 6.5 in the combination group, range 1-33) were needed to treat DME, and most injections were given during the first year of treatment. None of these patients received intravitreal corticosteroid or underwent vitrectomy.

In just over a third (37%) of the cases, there was only a single episode of DME during the follow-up period (Table 2). The recurrence of DME occurred in 91% of the cases in the observation group, 59% of the cases in the laser group, 65% of the cases in the anti-VEGF group and 37% of the cases in the combination group of laser and anti-VEGF during the average follow-up period of 65 months.

## Discussion

The duration of diabetes, poor glycemic control, high blood pressure and proteinuria are known risk factors when observing the development of diabetes related complications [11]. Globally, DR has been one of the leading causes of blindness among the working age population and the most common complication of T1D [12]. However, the amount of visual impairment due to DR has decreased during the past decades [7, 13]. More effective screening for DR and improvement in the management of both diabetes and DR have been key factors in the reduction of visual impairment [7]. Accordingly, DME related visual impairment was rare, and reduced in the present study during the 15-year follow-up in the population-based cohort of T1D patients with DME. We might assume that the improvements in both diagnostic accuracy and treatment possibilities of DME during the past 15 years have played an important role in this phenomenon.

Warwick et al. has reported that DME is associated with the duration of diabetes [3]. In agreement with this, our results showed that in T1D patients DME developed almost 10 years later than the first signs of DR. One may speculate that the prevalence of DME increases along the severity of DR. However, only less than one fourth of the study patients with DME had PDR. Previous study has revealed a high 94% prevalence of any DR and 35% prevalence of PDR in patients with T1D since childhood and duration of T1D for over twenty years [14]. The T1D patients with DME in the present study differ from this cohort of patients with T1D since childhood, according to older age at the onset of diabetes and the lower prevalence of PDR.

The treatment for DME has revolutionized during the last 15 years after the introduction of intravitreal anti-VEGF agents [7, 15]. Laser photocoagulation has been the standard treatment for DME for almost three decades, but currently anti-VEGF agents are considered as the first line treatment alternative in center-involving DME [8]. Several large clinical trials have demonstrated that the improvement of BCVA >15 ETDRS letters has been achieved by anti-VEGF agents in 33-45% of patients with either type 1 or type 2 diabetes [8, 16, 17]. Superior visual outcomes have been reported by ranibizumab treatment compared to treatment performed by laser, and a higher proportion of patients has gained significant >10-15 ETDRS letter increase in BCVA when treated with anti-VEGF [6]. Similarly, the BOLT study has revealed that the number of DME patients gaining >15 ETDRS letters was significantly greater when treated with bevacizumab compared to macular laser [18, 19]. In agreement with these results, DME patients gaining the most ETDRS letters in the current real-world study were treated with bevacizumab, and the sustained long-term visual outcomes were achieved with combination treatment of both anti-VEGF and macular laser. However, previous studies have suggested that DME patients with relatively good BCVA could be observed when BCVA remains stable [20–22]. Our real-world results show, however, a slight decrease in BCVA in the observation group of patients receiving no treatment in the long term, suggesting that the early treatment of DME might be reasonable to maintain good vision. This is of particular importance when considering the necessity of functional vision for these working-aged, relatively young patients with T1D. Accordingly, the benefits of early intensive treatment with anti-VEGF has recently been shown to result in satisfying long-term visual outcomes [23]. Even in the cases of no BCVA improvement, anti-VEGF treatment may improve contrast sensitivity in DME patients [20], and thus provide the best long-time safety in maintaining good visual function.

Beyond VEGF, the presence of inflammation is known to affect DME pathogenesis and intravitreal corticosteroids may be used to treat DME [8]. In our T1D patient cohort none of the patients received intravitreal corticosteroids. This might be explained by the known side-effects of corticosteroids, which include conditions such as cataract development or possible increase in intraocular pressure [24]. Most study patients, at the average age of 47 years, might be assumed not to have cataract decreasing visual acuity, and the formation of cataract might thus be attempted to avoid by primarily using anti-VEGF drugs classified with a more positive safety profile.

There are some limitations in our study. First, the retrospective nature of the study might affect the assembly of different treatment groups and thus comparison of the outcomes. Understandably, baseline visual acuities varied according to the presence or lack of central-involving edema in the participants. In addition, there was no defined BCVA-level for the choice of each treatment. Taking this into consideration, the intra- but no intergroup analysis in the long-term changes in BCVA were completed. Secondly, the data of the current study did not include the IOL status. However, the average age of the T1D patients in the study cohort was 47.4土14.4 years, and despite the presence of T1D formation of cataract in this age is not common. In addition, the underlying risk factors for DME were only partly available, and a precise data of the blood glucose levels, cholesterol and kidney function were lacking. Thirdly, the current data did not include any patients treated with intravitreal corticosteroids and the visual outcomes of that treatment cannot be concluded. We consider the inclusion of only T1D patients with DME as part of the current study as a strength in contrast to most studies that are completed with patients with both type 1 and type 2 diabetes. There are significant differences in the pathogenesis as well as risk factors for DME between these patient groups. Combining these patient groups might be a possible source of bias. Also, the real-world setting and long-term follow-up of the population-based cohort might be considered as strengths of the present study.

In conclusion, our results suggest that treatment of central DME by intravitreal anti-VEGF alone or in combination with laser is beneficial in terms of visual improvement in patients with T1D. A low rate of DME recurrences and the beneficial effect on contrast sensitivity [20, 24] highlights the importance of timely anti-VEGF in maintaining good visual function in patients with DME.

## Conclusions

During the past 15 years, anti-VEGF agents have become the first-line treatment for center-involving DME after laser photocoagulation being the golden standard treatment for DME for almost three decades. Though the benefits of anti-VEGF agents are well-known, the knowledge of long-term visual prognosis of patients with T1D and DME is still lacking.

This study reports, for the first time, long-term, real-world DME treatment outcomes solely in patients with T1D since the clinical features and risk factors for DME in T1D differ from those of T2D. Anti-VEGF alone, or in combination with macular laser, seems to improve visual outcomes and treatment stability in T1D patients with DME.

## Data Availability

The datasets generated during and analyzed during the current study are not publicly available due to confidentiality of data-protected patient information but are available from the corresponding author on reasonable request.
